# Potential use of EGFR-targeted molecular therapies for tumor suppressor CYLD-negative and poor prognosis oral squamous cell carcinoma with chemoresistance

**DOI:** 10.1186/s12935-022-02781-x

**Published:** 2022-11-15

**Authors:** Ayumi Kanemaru, Satoru Shinriki, Mimi Kai, Kanae Tsurekawa, Kazuya Ozeki, Shota Uchino, Naoki Suenaga, Kou Yonemaru, Shunsuke Miyake, Takeshi Masuda, Ryusho Kariya, Seiji Okada, Hisashi Takeshita, Yuki Seki, Hiromu Yano, Yoshihiro Komohara, Ryoji Yoshida, Hideki Nakayama, Jian-Dong Li, Hideyuki Saito, Hirofumi Jono

**Affiliations:** 1 Department of Clinical Pharmaceutical Sciences, Graduate School of Pharmaceutical Sciences, Kumamoto University, 2-2-1 Honjo, Chuo-Ku, Kumamoto, 860-0811 Japan; 2 Department of Molecular Laboratory Medicine, Faculty of Life Sciences, Kumamoto University, 1-1-1 Honjo, Chuo-Ku, Kumamoto, 860-8556 Japan; 3 Department of Pharmacy, Kumamoto University Hospital, 1-1-1 Honjo, Chuo-Ku, Kumamoto, 860-8556 Japan; 4 Department of Pharmaceutical Microbiology, Faculty of Life Sciences, Kumamoto University, 5-1 Oe honmachi, Chuo-Ku, Kumamoto, 862-0973 Japan; 5 Division of Hematopoiesis, Joint Research Center for Human Retrovirus Infection, Kumamoto University, 2-2-1 Honjo, Chuo-ku, Kumamoto, 860-0811 Japan; 6 Department of Oral & Maxillofacial Surgery, Faculty of Life Sciences, Kumamoto University, 1-1-1 Honjo, Chuo-Ku, Kumamoto, 860-8556 Japan; 7 Department of Cell Pathology, Faculty of Life Sciences, Kumamoto University, 1-1-1 Honjo, Chuo-Ku, Kumamoto, 860-8556 Japan; 8 Center for Inflammation, Immunity and Infection, Institute for Biomedical Sciences, Georgia State University, Atlanta, GA 30303 USA

**Keywords:** Cylindromatosis (CYLD), Epidermal growth factor receptor (EGFR) targeted molecular therapy, Oral squamous cell carcinoma (OSCC), Cytotoxic-chemotherapeutic resistance

## Abstract

**Background:**

Tumor suppressor CYLD dysfunction by loss of its expression, triggers malignant transformation, especially drug resistance and tumor invasion/metastasis. Although loss of CYLD expression is significantly associated with poor prognosis in a large variety of tumors, no clinically-effective treatment for CYLD-negative cancer patients is available.

**Methods:**

We focused on oral squamous cell carcinoma (OSCC), and sought to develop novel therapeutic agents for CYLD-negative cancer patients with poor prognosis. CYLD-knockdown OSCC cells by using CYLD-specific siRNA, were used to elucidate and determine the efficacy of novel drug candidates by evaluating cell viability and epithelial-mesenchymal transition (EMT)-like change. Therapeutic effects of candidate drug on cell line-derived xenograft (CDX) model and usefulness of CYLD as a novel biomarker using patient-derived xenograft (PDX) model were further investigated.

**Results:**

CYLD-knockdown OSCC cells were resistant for all currently-available cytotoxic chemotherapeutic agents for OSCC, such as, cisplatin, 5-FU, carboplatin, docetaxel, and paclitaxel. By using comprehensive proteome analysis approach, we identified epidermal growth factor receptor (EGFR), a receptor tyrosine kinase, played key roles in CYLD-knockdown OSCC cells. Indeed, cell survival rate in the cisplatin-resistant CYLD-knockdown OSCC cells was markedly inhibited by treatment with clinically available EGFR tyrosine kinase inhibitors (EGFR-TKIs), such as gefitinib. In addition, gefitinib was significantly effective for not only cell survival, but also EMT-like changes through inhibiting transforming growth factor-β (TGF-β) signaling in CYLD-knockdown OSCC cells. Thereby, overall survival of CYLD-knockdown CDX models was significantly prolonged by gefitinib treatment. Moreover, we found that CYLD expression was significantly associated with gefitinib response by using PDX models.

**Conclusions:**

Our results first revealed that EGFR-targeted molecular therapies, such as EGFR-TKIs, could have potential to be novel therapeutic agents for the CYLD-negative OSCC patients with poor prognosis.

**Supplementary Information:**

The online version contains supplementary material available at 10.1186/s12935-022-02781-x.

## Background

Cylindromatosis (CYLD) gene was originally discovered as a tumor suppressor gene, that has been identified as the causative gene for familial cylindromatosis [1]. CYLD has shown to serve as deubiquitinase and inhibit activity of target molecules by removing lysine-63 (K63)-mediated polyubiquitin chains, which are involved in cell signal transduction [2]. Molecular biological analysis identified the target molecules for CYLD, such as, tumor necrosis factor receptor-associated factor (TRAF)2, TRAF6, and nuclear factor-κB (NF-κB) essential modulator (NEMO), which are necessary elements for NF-κB activation [3–5]. Since NF-κB activation has an anti-apoptotic effect, CYLD dysfunction indeed lead to cell immortalization and tumorigenesis due to NF-κB hyper-activation. Subsequent researches reported that CYLD targeted and regulated a variety of signaling pathways [6–13], and revealed that loss of CYLD function might play various and pivotal roles in a variety of diseases caused by abnormal intracellular signaling, especially in malignant tumors [2].

Besides the studies showing many different types of CYLD mutations involved in tumorigenesis [14], recent clinical studies for a variety of tumors reveal that, CYLD dysfunction due to loss of its protein expression, rather than mutations, is a crucial prognostic factor for both malignant transformation and poor prognosis in various tumors. Massoumi et al. revealed that, in malignant melanoma, loss of CYLD expression promoted tumor progression via increased tumor cell proliferation and migration, and patients with lower CYLD expression exhibited significantly shorter overall survival [15]. Moreover, significant amounts of clinical study have demonstrated the loss of CYLD expression in a variety of malignant tumors, such as, hepatocellular carcinoma [16, 17], melanoma [18], basal cell carcinoma [19], breast cancer [20], glioblastoma [21], and cholesteatoma [22]. Those studies revealed that loss of CYLD expression was significantly associated with poor overall survival, and unveiled the pathogenesis of malignant transformation in CYLD-negative cancer patients. However, those studies also revealed that loss of CYLD expression was closely associated with drug resistance against multiple anticancer drugs in many types of cancers [17, 23, 24]. For instance, in hepatocellular carcinoma (HCC), CYLD expression was down-regulated and involved in the resistance towards treatment with doxorubicin, 5-FU, and cisplatin [17]. Thus, despite the urgent need for developing novel therapeutic strategies for CYLD-negative cancer patients with poor prognosis, effective treatment in clinical has not been established yet, as of this moment.

Oral cancer is one of the most common malignancies in head and neck carcinoma, of which more than 90% of oral cancer are oral squamous cell carcinoma (OSCC) [25]. Despite recent advances in early diagnosis and treatment, the 5-year survival rate for patients with OSCC has not improved [26]. Although surgical therapy is recommended as the first-line treatment, OSCC is highly invasive and carries the risk of compromising the patient's quality of life, suggesting that the development of minimally invasive non-surgical pharmacotherapy is highly desirable. However, although chemotherapeutic agents, such as an alkylating agent cisplatin (*cis*-diamminedichloroplatinum II), is a useful treatment for advanced OSCC patients, development of intrinsic or acquired cisplatin resistance is main responsible factor for poor overall survival [27]. Thus, understanding the features and mediators of OSCC invasion and drug resistance is of critical importance for the development of new treatment approaches. In particular, our previous clinical study uncovered that loss of CYLD expression in OSCC tissues was significantly associated with the clinical features of deep invasion and poor overall survival of OSCC patients [28]. In OSCC, down-regulation of CYLD expression significantly enhanced tumor cell invasion through epithelial-mesenchymal transition (EMT)-like changes through promoted transforming growth factor-β (TGF-β) signaling. Moreover, loss of CYLD expression might cause cisplatin resistance through NF-κB hyper-activation, which in turn, lead to poor prognosis in CYLD-negative OSCC patients [29]. While those studies strongly suggest that loss of CYLD expression is a crucial factor determining the poor prognosis of OSCC patients, a novel therapeutic target and agent for CYLD-negative OSCC patients has yet to be identified.

Here, we sought to provide novel treatments for CYLD-negative cancer patients with poor prognosis, and performed multiple experimental approaches to identify the crucial cell signaling pathway responsible for CYLD-dependent malignant transformation. Our results first showed that epidermal growth factor receptor (EGFR), a receptor tyrosine kinase, served as a novel therapeutic target, and that EGFR-targeted molecular therapies, including EGFR tyrosine kinase inhibitors (EGFR-TKIs) and monoclonal antibody, could be effective for CYLD-negative OSCC cells with chemoresistance.

## Methods

### Antibodies and reagents

Rabbit polyclonal anti-CYLD antibody (SAB4200060) was purchased from Sigma-Aldrich, Inc (Saint Louis, MO, USA). Rabbit monoclonal anti-EGFR antibody (#4267), rabbit monoclonal anti-Phospho-EGFR antibody (#3777), rabbit monoclonal anti-Akt antibody (#4691S), rabbit monoclonal anti-Phospho-Akt antibody (#4060S), rabbit monoclonal anti-ERK antibody (#4695), rabbit monoclonal anti-Phospho-ERK antibody (#4370S), rabbit monoclonal anti-Smad2/3 antibody (#3102S), and rabbit monoclonal anti-Phospho-Smad3 antibody (#9520S) were purchased from Cell Signaling Technology (Beverly, MA, USA). PI3K inhibitor (LY294002, HY-10108), MEK inhibitor (PD98059, HY-12028), and ALK5 inhibitor (TGF-β RI Kinase Inhibitor II, #616452) were purchased from Calbiochem (San Diego, CA, USA). Cisplatin (Nippon Kayaku, Tokyo, Japan), 5-FU (Sigma-Aldrich, Inc), cetuximab (Merck BioPharma, Darmstadt, Germany), gefitinib (Tocris Bioscience, Bristol, UK), erlotinib (Wako, Tokyo, Japan), afatinib (abcam, Cambridge, UK), and osimertinib (Selleck Chemicals, Houston, USA) were also used in this study. For other reagents, commercially available special grades were used.

### Cells lines and cell culture

Human OSCC cell lines SAS (TKG 0470, Cell Resource Center for Biomedical Research, Cell Bank, Tohoku University) were used in this study. SAS cells were grown in RPMI 1640 (Thermo Fisher Scientific, Waltham, MA, USA) with 10% heat-inactivated fetal bovine serum (Thermo Fisher Scientific) in 5% CO_2_ at 37 ℃.

### Transfection with siRNA

SAS cells were incubated in 12-well plates (0.8 × 10^5^ cells/mL) for 24 h and were transiently transfected with siRNA (50 nM) by using Lipofectamine 2000 (Thermo Fisher Scientific) according to the manufacturer’s protocol. After transfection and incubation for 48 h, experiments were performed (Additional file 1: Fig. S1). Silencer Negative Control siRNA (Ambion/Applied Biosystems, Foster City, CA) was used as the control (siN). Sequences of siRNA targeting CYLD (siCYLD) were sense: 5′-GAUUGUUACUUCUAUCAAAtt-3′ and antisense: 5′-UUUGAUAGAAGUAACAAUCtt-3′.

### Cell viability assay

Cells were incubated in 96-well plates and treated with various anticancer agents (cisplatin: 0.1–10 µg/mL, 5-FU: 0.01–10 µg/mL, carboplatin: 1–500 µM, docetaxel: 0.1–1000 ng/mL, paclitaxel: 10–1000 nM, gefitinib: 0.01–50 µM, erlotinib: 0.1–18 µM, afatinib: 0.001–25 µM, osimertinib: 0.01–50 µM, cetuximab: 0.1–250 µg/mL) in serum-free RPMI 1640. After incubation, 10 µL/well Cell Counting Kit-8 solution (DOJINDO LABORATORIES, Kumamoto, Japan) were added to the cells and measured the absorbance at 450 nm by EMax SOFTmaxPRO (molecular devices, Tokyo, Japan).

### RNA isolation and quantitative real-time PCR (RT-qPCR)

Total RNA extraction from cells was performed by phenol chloroform extraction method using TRIzol (Invitrogen, Waltham, Massachusetts, USA). Total RNA (0.5 µg) was reverse-transcribed to cDNA by the PrimeScript RT reagent (Takara Bio Inc, Shiga, Japan) according to the manufacturer’s instructions. For quantification of mRNA, each PCR assay was performed using 1 µL of cDNA and each primer (10 µM) 0.3 µL, in a LightCycler System with SYBR Premix DimerEraser (Takara Bio Inc), with each reaction (with 10 µL samples) was performed under following conditions: polymerase activation at 95 ℃ for 30 s followed by 40 cycles of and 95 ℃ for 5 s, 55 ℃ for 30 s, 72 ℃ for 30 s. Human 18S ribosomal RNA (rRNA) was used as an internal control. Relative expression levels were determined by the ΔΔCt method. The sequences of each primer were as follows: Human CYLD sense: 5′-TCAGGCTTATGGAGCCAAGAA-3′, antisense: 5′-ACTTCCCTTCGGTACTTTAAGGA-3′; human 18s rRNA sense: 5′-CGGCTACCACATCCAAGGAA-3′, antisense: 5′-GCTGGAATTACCGCGGCT-3′; Human Fibronectin sense: 5′-CAGTGGGAGACCTCGAGAAG-3′, antisense: 5′-TCCCTCGGAACATCAGAAAC-3′.

### Protein extraction and immunoblotting

Cells were washed once with ice-cold PBS and then lysed by adding RIPA Buffer (1×) with 1 mM PMSF (Cell Signaling Technology). Supernatants were stored at − 80 ℃ until use. Equal amounts of protein were fractionated via sodium dodecyl sulfate polyacrylamide gel electrophoresis and transferred to PVDF membranes (Millipore, Massachusetts, USA). Membranes were blocked with 5% non-fat dried milk (Cell Signaling Technology) in tris-buffered saline (0.05 M Tris, 0.138 M NaCl, 0.0027 M KCl, pH 7.8) containing 0.1% Tween-20 (TBS-T) and were then incubated overnight at 4 °C with antibodies primary antibody in TBS-T containing 2% nonfat dry milk or 5% bovine serum albumin (Sigma Aldrich). After membranes were washed with TBS-T, they were incubated for 1 h in horseradish peroxidase (HRP)-conjugated secondary antibodies at room temperature. After washing, specific protein bands were detected by using ECL Prime Western Blotting Detection Reagents (Amersham Life Science, Arlington Heights, IL, USA), according to the manufacturer’s instructions.

### Proteome analysis by liquid chromatograph-mass spectrometry (LC–MS/MS)

Whole cell lysate of SAS cells was prepared by phase transfer surfactant (PTS) method as described previously [30, 31]. Sodium deoxycholate (SDC), sodium *N*-lauroylsarcosinate (SLS), ammonium bicarbonate, dithiothreitol, iodoacetamide, mass spectrometry grade lysyl endoprotease, ethyl acetate, acetonitrile, acetic acid, methanol, trifluoroacetic acid (Wako, Osaka, Japan), modified trypsin (Promega, Madison, MA), and 4-(2-Aminoethyl) benzenesulfonyl fluoride hydrochloride (Nacalai, Kyoto, Japan) were used. Proteins were extracted with PTS solution (12 mM SDC, 12 mM SLS, and 100mM Tris-HCl (pH9.0) and ultrasonic crushed for 20 min. After incubating for 5 min at 95 ℃, proteins in the supernatant solution were quantified by the BCA method using BCA Protein Assay Kit (Thermo Fisher Scientific). Proteins were reduced with 10 mM dithiothreitol for 30 min and alkylated with 50 mM iodoacetamide in the dark for 30 min at room temperature. The protein mixture was twofold diluted with 50 mM ammonium bicarbonate. And then, mixture was digested with Lys-C (1/50 sample weight) at room temperature for 3 h prior to the addition of trypsin (1/50 sample weight) and incubated at 37 ℃ for 20 h. An equal volume of ethyl acetate was added to the sample solution, and the mixture was acidified with 0.5% trifluoroacetic acid (final concentration). The mixture was shaken for 2 min and centrifuged at 15,600×*g* for 2 min, and then the upper layer was removed by pipette, and dry up by a vacuum evaporator. Suspending the sample in 100 μL buffer A (5% acetonitrile, 0.1% TFA) and desalt with GL-Tip SDB (GL Science, Tokyo, Japan) [32, 33]. A TripleTOF 5600 (SCIEX, Framingham, MA, USA) equipped with a Dionex Ultimate 3000 RSLS (Thermo Fisher Scientific) was employed for nanoLC-MS/MS measurement. The injection volume was 5 µL, and the flow rate was 300 nL/min. A nano-trap column (100 µm ID, 2 cm length, packed with 5 µm Acclaim PepMap100C18, Thermo Fisher Scientific) and an analytical nanocolumn (75 µm ID, 25 cm length, packed with 2 µm Acclaim PepMap C18, Thermo Fisher Scientific) were used. The MS data was acquired with Analyst Software TF 1.7 (SCIEX). For peptide identification, data were acquired in the data-dependent acquisition mode and analyzed using ProteinPilot 4.5 (SCIEX) connected to the UniProt human reference proteome database (release 2017_11). For protein and peptide quantification, data were acquired in the data-independent acquisition mode (Sequential window acquisition of all theoretical fragment ion spectra: SWATH-MS) with a variable window for the precursor ions [34]. The results were analyzed with Enrichr (https://maayanlab.cloud/Enrichr/), by which and enriched pathways included in KEGG (Kyoto Encyclopedia of Genes and Genomes) 2021 human database were identified. Among the top 50 enriched pathways, those related to signal transduction (3.2 signal transduction; https://www.genome.jp/kegg/pathway.html#environmental) were extracted and listed in order of p-value.

### Apoptosis assay

Cells were stained with PE Annexin V Apoptosis Detection Kit I (BD Biosciences, Tokyo, Japan), according to the manufacturer’s protocol, and apoptosis was measured using the FACS Verse Flow Cytometer (BD Biosciences).

### Wound healing assay

After knocking-down CYLD expression in SAS cells, the medium was changed to serum-free RPMI 1640. After culturing for 24 h, cells were scratched with a 200 µL micropipette tip. At the same time as scratching, cells were treated with gefitinib and cetuximab in serum-free RPMI1640. After 8 h, the percentage of the healed area was evaluated using Image J software (National Institutes of Health, Bethesda, MD, USA).

### Transwell migration assay

After knocking-down CYLD expression, SAS (1.0 × 10^5^) cells were suspended in serum-free RPMI 1640 and seeded in upper part of Transwell insert (8 µm-filters, Corning, New York, USA). Cells migration was induced by RPMI 1640 containing 10% heat-inactivated fetal bovine serum. After 24 h, the migrating cells were stained by crystal violet, and the ratio of the stained area was quantified by Image J software.

### Generation of tumor cell line-derived xenograft (CDX) model and gefitinib treatment

Male CB17/ICR-scid/scid mice (SCID mice), each 7–8 weeks old and weighting 20 to 25 g, were obtained from CLEA Japan and maintained in a specific pathogen-free environment at the Center for Animal Resources and Development, Kumamoto University. SAS cells were trypsinized, washed with 10% heat-inactivated fetal bovine serum RPMI 1640, resuspended in PBS, and adjusted to a concentration of 1 × 10^7^ cells/100 μL in PBS. Then the cell suspensions were injected 100 µL/mice subcutaneously into SCID mice. After 7 days from the cell injection, the CDX mice were injected intraperitoneally with 50 µL DMSO containing 2.5 mg of gefitinib or DMSO alone and received the same treatment for 5 times on days 7–11. Tumor development was followed in individual animals every 3–4 days by sequential caliper measurements of length (L) and width (W). Tumor volume was calculated by the formula LW^2^ π/6. The Animal Care and Use Committee of Kumamoto University approved the protocols for all animal experiments.

### Analyses of patient-derived xenograft (PDX) tumors and PDX-derived cell lines

PDX-model strains (PDX st.1 and PDX st.2) and corresponding PDX-derived cell lines were prepared as described previously [35, 36]. This study has been approved by the ethical committee at Kumamoto University (approval No. 1427, No. 2389). The cells were grown in DMEM (Thermo Fisher Scientific) with 10% heat-inactivated fetal bovine serum (Thermo Fisher Scientific) in 5% CO_2_ at 37 ℃.

### Immunohistochemistry

Tissue samples for immunohistochemistry were fixed with 10% formalin before processing. Formalin-fixed specimens of clinical tissues were embedded in paraffin, cut into 5-μm-thick sections, and mounted on slides. These sections were dewaxed in xylene and then rehydrated in descending concentrations of alcohol. Endogenous peroxidase was blocked via a 30 min incubation of slides with 3% hydrogen peroxide. After slides were washed with PBS for 5 min, a non-specific staining blocking reagent (Nacalai tesque) was used for 10 min to block non-specific background staining, followed by overnight incubation at 4 ℃ with antibody against CYLD (1:50) diluted in PBS containing 1% BSA. Slides were rinsed with PBS for 5 min, incubated with secondary antibodies for 1 h, and washed again with PBS, and incubated with Liquid DAB Substrate Chromogen System (DAKO, Glostrup, Denmark) according to the manufacturer’s protocol. Slides were lightly counterstained with haematoxylin for 30 s before dehydration and mounting. Three persons took 10 photos per tissue in a blind state. CYLD protein expression levels in a total of 30 photos per tissue were quantified using ImageJ software.

### Statistical analysis

Tukey–Kramer method were used to evaluate differences between more than three groups. Data are represented as the mean ± standard deviation (S.D.). The Mann–Whitney U test were used to assess the differences between the two groups that were not normally distributed. P-values of < 0.05 were said to be statistically significant.

## Results

### Sensitivity of chemotherapeutic agents to CYLD-knockdown OSCC cells

**Fig. 1:**
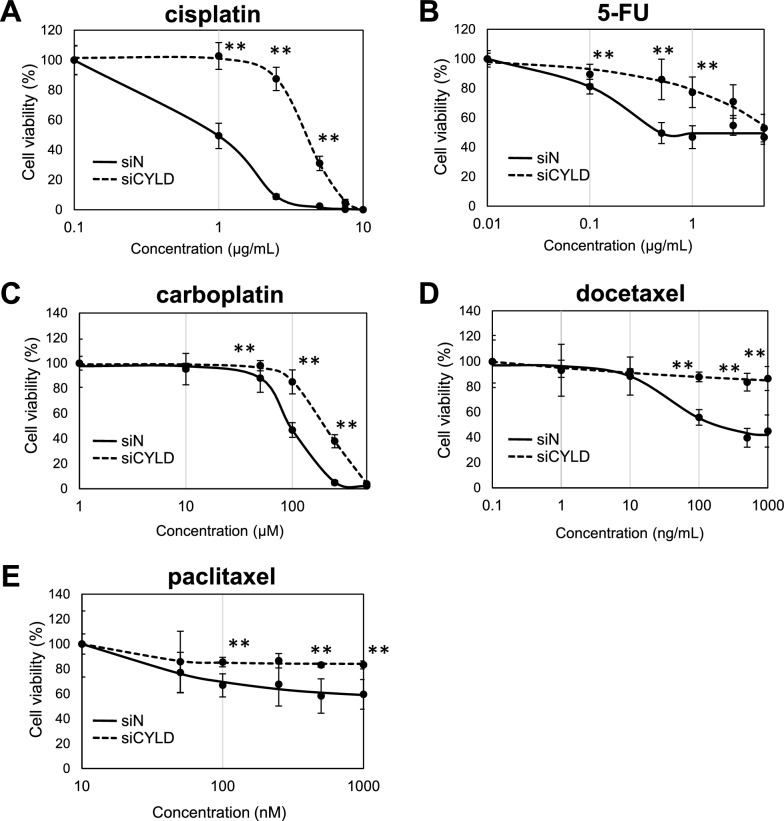
Sensitivity of chemotherapeutic agents to CYLD-knockdown OSCC cells. Human OSCC cell line (SAS) cells were transfected with control siRNA (siN) or CYLD-specific siRNA (siCYLD) and then treated with cisplatin (**A**), 5-FU (**B**), carboplatin (**C**), docetaxel (**D**), and paclitaxel (**E**). The cell survival rates of SAS cells were assessed 72 h after treatment. Values are means ± S.D. of quadruple samples. **p < 0.01 vs siN group in Tukey–Kramer method

Although the previous clinical study has shown that loss of CYLD expression is significantly associated with poor overall survival, as of moment, clinically-effective treatments for CYLD-negative OSCC patients has not been established [28]. We first sought to find the effective anti-tumor drugs for CYLD-knockdown OSCC cells, among the chemotherapeutic agents currently approved for standard treatment for OSCC patients [37, 38]. For CYLD-knockdown OSCC cells, sensitivities to chemotherapeutic agents, such as, cisplatin, 5-FU, carboplatin, docetaxel, and paclitaxel, were evaluated by the cell viability assay. As shown in Fig. 1, the CYLD-knockdown OSCC cells exhibited higher cell viability for all chemotherapeutic agent than cells transfected with control siRNA (siN), suggesting that CYLD-knockdown OSCC cells were resistant for all currently-available standard treatment, especially the cytotoxic chemotherapeutic agents.

### Effect of CYLD down-regulation on intracellular signaling pathways in OSCC cells

**Fig. 2:**
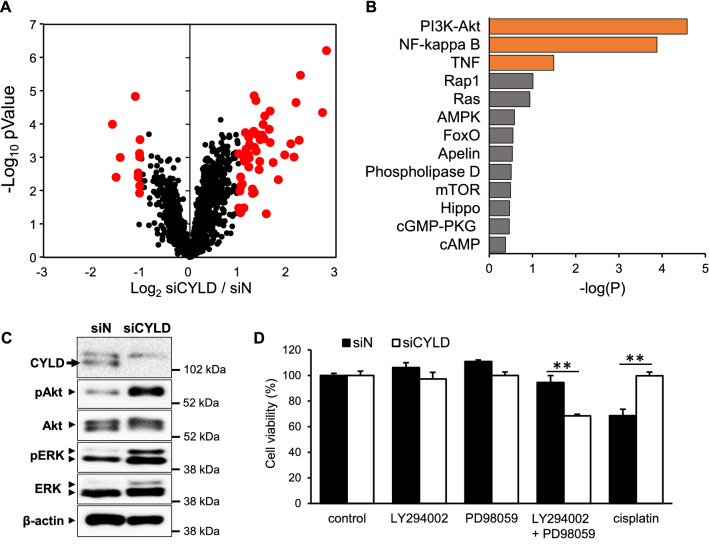
Effect of CYLD down-regulation on intracellular signaling pathways in OSCC cells. **A** Comprehensive changes in protein expression by CYLD knockdown were assessed by proteome analysis. **B** KEGG database analysis of cell signaling pathways activated by CYLD knockdown. **C** Phosphorylation of Akt and ERK was assessed by immunoblotting. Cells were transfected with the indicated siRNAs and then incubated for 72 h before harvesting. Cell lysate was immunoblotted with antibodies against the indicated proteins. **D** Cells were treated with LY294002 (Akt Inhibitor, 20 µM), PD98059 (ERK Inhibitor, 20 µM), combination, and cisplatin (8 µg/mL). Cells survival rate are assessed after 72 h. Values are means ± S.D. of triplicate samples. **p < 0.01 in Tukey–Kramer method

It has been reported that CYLD is involved in the pathogenesis of tumor progression via regulating a variety of intracellular signaling pathways as a deubiquitinating enzyme [2, 3, 12, 17]. Because of no conventional treatment, we next investigated the effect of CYLD down-regulation on intracellular signaling pathways and attempted to identify novel therapeutic targets for CYLD-knockdown OSCC cells. We performed proteome analysis to comprehensively identify intracellular signaling pathways activated in CYLD-knockdown OSCC cells. The results in Fig. 2A indicating the global protein expression alterations, suggested there were 49 proteins increased in OSCC cells more than doubled by CYLD knockdown. In addition, KEGG analysis demonstrated that the protein sets associated with phosphatidylinositol-3 kinase (PI3K)-Akt signaling pathway was markedly enriched in CYLD-knockdown OSCC cells (Fig. 2B, Additional file 1: Fig. S2). Aberrant activation of the PI3K-Akt signaling pathway is known to have a significant role in carcinogenesis, alongside of critical roles of mitogen-activated protein kinase (MAPK) cascades in drug resistance and sensitivity [39, 40]. As shown in Fig. 2C, immunoblotting analysis confirmed that CYLD knockdown indeed increased phosphorylation of Akt, and also phosphorylation of ERK, one of signal molecule in MAPK signaling. We further determined whether these cell signaling pathways enhanced by CYLD knockdown were involved in the cell viability of OSCC cells. As shown in Fig. 2D, although treatment of either Akt (LY294002) or ERK (PD98059) inhibitor alone was not effective on the cell viability, combined treatment significantly decreased the cell viability of CYLD-knockdown OSCC cells, in contrast to the observed cisplatin resistance. These results suggested that the intracellular signaling pathways activated in CYLD-knockdown OSCC cells, might be the novel therapeutic targets, and simultaneous inhibition of Akt and ERK could be effective for CYLD-knockdown OSCC cells.

### Involvement of EGFR in cell viability of CYLD-knockdown OSCC cells

**Fig. 3:**
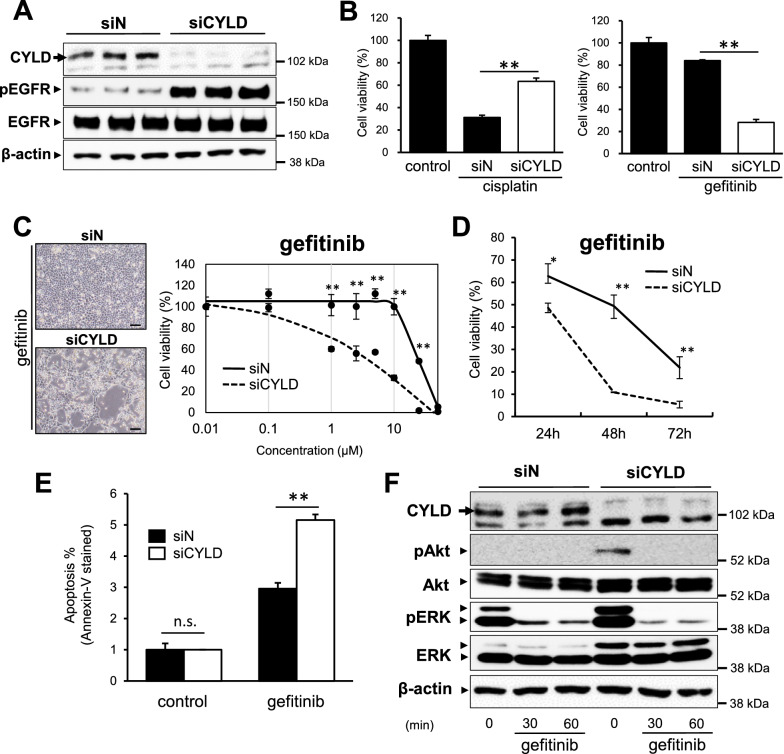
Involvement of EGFR in cell viability of CYLD-knockdown OSCC cells. **A** EGFR phosphorylation was assessed by immunoblotting in OSCC cells. Cells were transfected with siRNA and then incubated for 72 h before harvesting. Cell lysate was immunoblotted with antibodies against the indicated proteins. **B** Cells were treated with gefitinib (10 µM) and cisplatin (8 µg/mL), and cells survival rate was assessed after 72 h. Relative cell survival rate of siCYLD cells compared to that of siN cells after treatment with each anticancer drug was shown. Values are means ± S.D. of triplicate samples. **p < 0.01 in Tukey–Kramer method. **C** (left panels) Representative images of cells treated with 10 µM gefitinib for 72 h. Scale bars show 100 µm. (right panel). Cell survival rates of OSCC cells were evaluated 72 h after treatment with various concentrations of gefitinib. Values are means ± S.D. of triplicate samples. **p < 0.01 vs siN group in Tukey–Kramer method. **D** Cells were treated with gefitinib (10 µM) and the cell survival rates were evaluated 24–72 h after treatment. Values are means ± S.D. of triplicate samples. * p < 0.05, ** p < 0.01 vs siN group in Tukey–Kramer method. (E) The gefitinib (10 µM)-induced apoptosis was assessed by Annexin-V/7-AAD staining using flow cytometry. Values are means ± S.D. of triplicate samples. n.s.: not significant, **p < 0.01 vs siN group in Tukey–Kramer method. **F** Phosphorylation of Akt and ERK were assessed by immunoblotting in OSCC cells treated with 10 µM gefitinib

EGFR, a receptor tyrosine kinase also known as common upstream kinase of both Akt and ERK signalings, plays key roles in cell growth and differentiation pathways in head and neck squamous cell carcinoma (HNSCC) [41]. Thus, we next focused on the involvement of EGFR in the cell viability of CYLD-knockdown OSCC cells. As shown in Fig. 3A, EGFR phosphorylation was markedly increased by CYLD knockdown. To further determine the involvement of EGFR phosphorylation in CYLD-knockdown OSCC cells, the cell viability was assessed by treatment with gefitinib, a well-known EGFR tyrosine kinase inhibitor (EGFR-TKI) [42, 43]. Notably, the cell survival rate in the cisplatin-resistant CYLD-knockdown OSCC cells was significantly decreased by even the gefitinib treatment alone (Fig. 3B, Additional file 1: Fig. S3). The effects of gefitinib on cell survival were indeed confirmed in a dose-dependent (Fig. 3C) and time-dependent (Fig. 3D) manner. In CYLD-knockdown OSCC cells, apoptotic cells were significantly increased by gefitinib treatment, compared with control cells (Fig. 3E). Consistent with the results shown in Fig. 2, the phosphorylation of two key kinases (Akt and ERK) caused by CYLD knockdown was significantly suppressed by gefitinib treatment alone (Fig. 3F). These results suggested that inhibiting the tyrosine kinase activity of EGFR, the upstream kinase of both Akt and ERK signalings, could be effective for CYLD-knockdown OSCC cells.

### Effect of EGFR-targeted inhibitors on cell viability of CYLD-knockdown OSCC cells

**Fig. 4:**
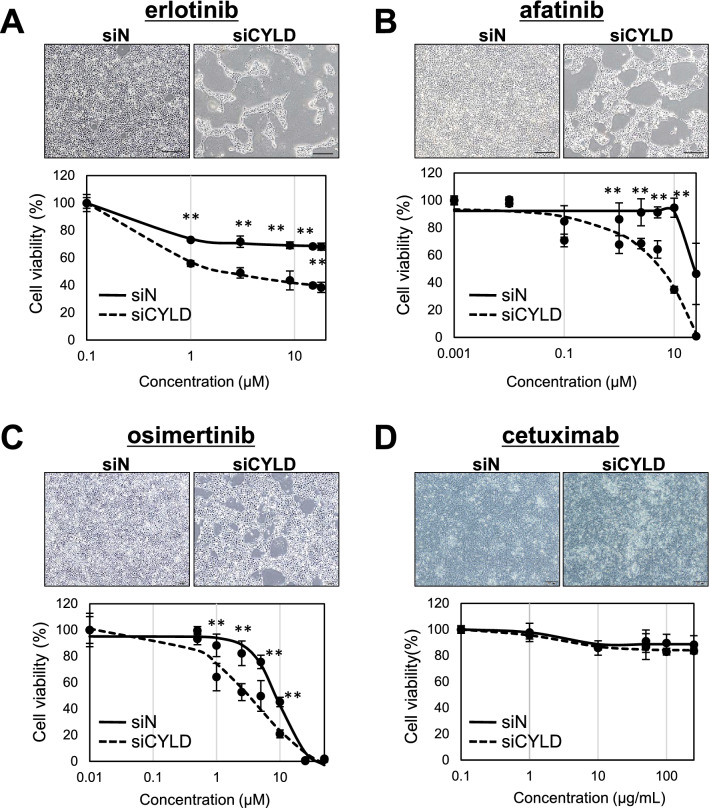
Effect of EGFR targeted inhibitors on cell viability of CYLD-knockdown OSCC cells. Cells were treated with erlotinib (**A**), afatinib (**B**), osimertinib (**C**) and cetuximab (**D**), and the cell survival rates were assessed 72 h after treatment. Values Scale bar shows 200 µm. Values are means ± S.D. of triplicate samples. **p < 0.01 vs siN group in Tukey–Kramer method

Several EGFR-targeted inhibitors, including EGFR-TKIs and monoclonal antibodies, are currently being developed and have been approved for clinical-use [44]. In addition to gefitinib, we also determined whether various EGFR targeted inhibitors were effective for CYLD-knockdown OSCC cells. As shown in Fig. 4A–C, it was notable that all EGFR-TKI, such as erlotinib, afatinib, and osimertinib, significantly decreased the cell viability of CYLD-knockdown OSCC cells, comparable with gefitinib treatment. In contrast, other EGFR-targeted inhibitor cetuximab, a human/murine chimeric IgG1 monoclonal EGFR antibody, was not effective for CYLD-knockdown OSCC cells, although cetuximab was the only EGFR-targeted inhibitor currently approved for OSCC treatment (Fig. 4D) [45]. These results suggested that various EGFR-TKIs could also be effective for the cell viability of CYLD-knockdown OSCC cells, in contrast to the observed chemoresistance.

### Effect of EGFR-targeted inhibitors on EMT-like changes in CYLD-knockdown OSCC cells

**Fig. 5:**
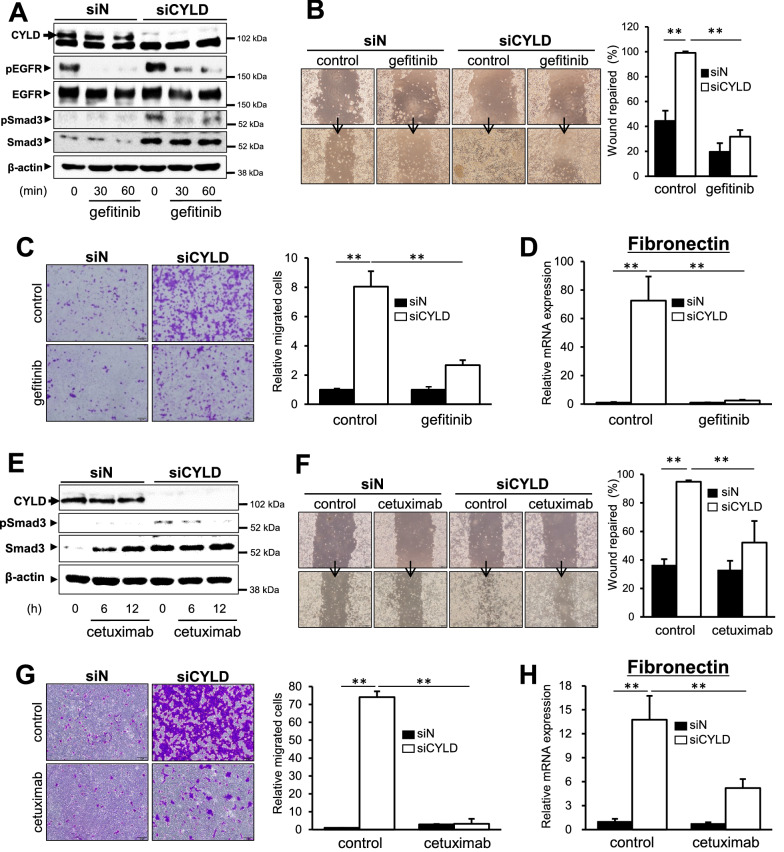
Effect of EGFR targeted inhibitors on EMT-like changes in CYLD-knockdown OSCC cells. (A, E) SAS cells were treated with 5 μM gefitinib (**A**) and 10 µg/mL cetuximab (**E**), and Smad3 phosphorylation were assessed by immunoblotting. (**B**, **F**) Cell migration in SAS cells treated with 5 μM gefitinib (**B**) and 10 µg/mL cetuximab (**F**) was assessed by wound healing assay. (**C**, **G**) Cells were transfected with siRNA and then incubated for 48 h before being reseeded onto transwell insert. Cells were treated with 0.15 μM gefitinib (**C**) and 10 µg/mL cetuximab (**G**) for 24 h, and migrating cells were stained by crystal violet and then measured. **D**, **H** CYLD knockdown cells were treated with 5 μM gefitinib (**D**) and 10 µg/mL cetuximab (**H**) for 24 h, and Fibronectin mRNA expression was assessed by RT-qPCR. Scale bar shows 200 µm. Values are means ± S.D. of triplicate samples. **p < 0.01 in Tukey–Kramer method

CYLD expression has shown to be associated with poor prognosis of OSCC patients via inducing the malignant transformation, such as EMT-like changes through TGF-β signaling [28]. We next determined whether EGFR-targeted inhibitors were also effective for the EMT-like changes induced in CYLD-knockdown OSCC cells. Regarding the activation of TGF-β signaling involved in EMT-like changes, CYLD knockdown-induced Smad3 phosphorylation was inhibited by gefitinib treatment (Fig. 5A). In addition, both wound healing assay (Fig. 5B) and transwell migration assay (Fig. 5C) showed that gefitinib treatment indeed suppressed cell migration caused by CYLD knockdown. Moreover, mRNA expression of Fibronectin, an EMT marker, was significantly suppressed by gefitinib, indicating that gefitinib was also effective for the EMT-like changes induced in CYLD-knockdown OSCC cells (Fig. 5D, Additional file 1: Fig. S4). These results suggested that gefitinib could be effective for not only cell survival, but also EMT-like changes through inhibiting TGF-β signaling in CYLD-knockdown OSCC cells. Interestingly, it should be noted that cetuximab also had potential to significantly inhibit Smad3 phosphorylation (Fig. 5E), cell migration (Fig. 5F, G) and Fibronectin expression (Fig. 5H) induced by CYLD knockdown, despite no effect on cell viability (Fig. 4D). Taken together, we could find novel effective drugs (gefitinib for inhibiting both cell survival and EMT-like changes, or cetuximab for inhibiting EMT-like changes) for CYLD-knockdown OSCC cells.

### Therapeutic effect of gefitinib and usefulness of CYLD as a novel biomarker in xenograft models

**Fig. 6:**
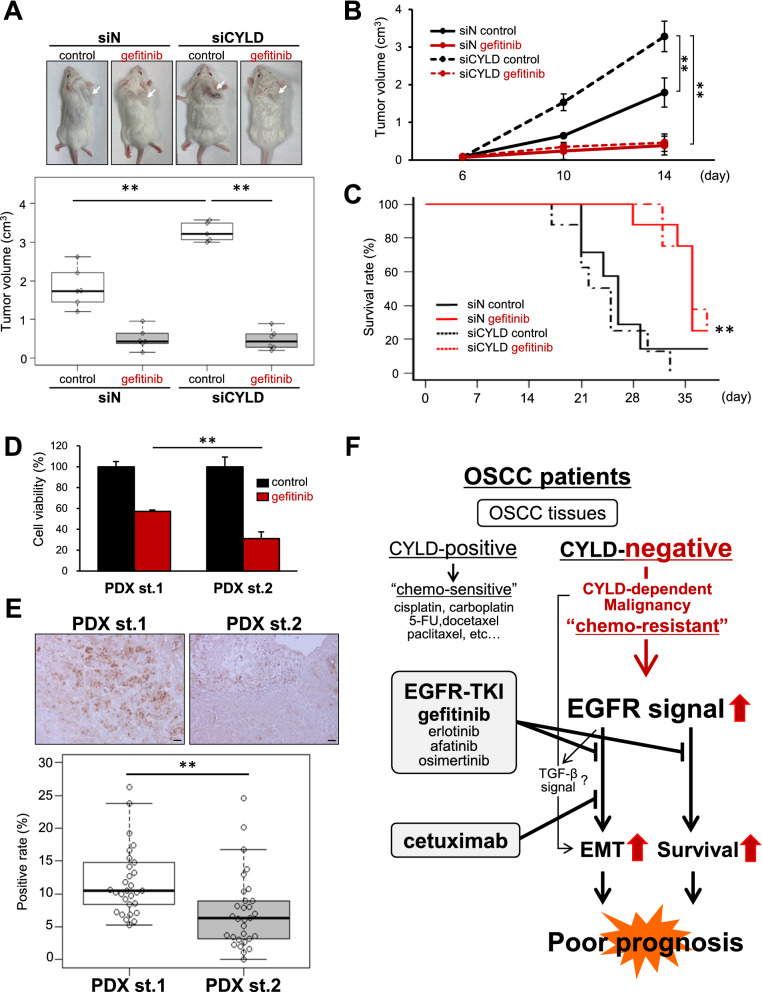
Therapeutic effect of gefitinib and usefulness of CYLD as a novel biomarker in xenograft models. **A**, **B** SAS cells were injected subcutaneously into SCID mice (n = 6). After 7 days from cells injection, the tumor-inoculated CDX models were injected intraperitoneally with 2.5 mg gefitinib or DMSO, and tumor volume were measured at 14 days (**A**) in a time-dependent manner (**B**). Values are means ± S.D. of triplicate samples. **p < 0.01 in Tukey Kramer test. **C** Kaplan–Meier plot of overall survival of CDX models. **p < 0.01 siCYLD control group vs siCYLD gefitinib group in log-rank test. **D** PDX-derived cells (PDX st.1 & PDX st.2) were treated with gefitinib, and the cell survival rates were assessed 48 h after treatment. Values Scale bar shows 200 µm. Values are means ± S.D. of triplicate samples. **p < 0.01 in Tukey–Kramer method. **E** Immunohistochemical analysis for CYLD expression in tumor tissues from PDX models (PDX st.1 & PDX st.2). CYLD positive rates were be calculated by ImageJ. Boxplots represented the first, second, and third quartiles, and whiskers extended to maximum value and minimum value except for outliers. Values Scale bar shows 40 µm. **p < 0.01 in Mann–Whitney U test. **F** Schematic model illustrating novel therapeutic strategies for CYLD-negative OSCC patients with poor prognosis

To further investigate the therapeutic effect in vivo, we first established a cell line-derived xenograft (CDX) model using OSCC cells. As shown in Fig. 6A, tumor volume was significantly increased in mice transplanted with CYLD-knockdown cells, compared with mice transplanted with control cells. In this CYLD-negative CDX models, gefitinib treatment significantly suppressed tumor growth during administration period (Fig. 6A, B). Moreover, overall survival of CYLD-negative CDX models, was significantly prolonged by gefitinib treatment (Fig. 6C), suggesting that the therapeutic effect of EGFR-TKI (gefitinib) treatment was indeed confirmed in CYLD-negative CDX models. Furthermore, patient-derived xenograft (PDX) models, generated by direct transplantation of patient tumor samples into immunocompromised mice, are recognized as the most relevant in vivo cancer models [46]. We finally generated PDX models from different patients and established PDX-derived cells from those models for determining whether CYLD might serve as a predictive biomarker for gefitinib treatment. Interestingly, we found that there were PDX-derived cells exhibiting either low sensitivity (PDX st.1) or high sensitivity (PDX st.2) for gefitinib treatment (Fig. 6D). We then confirmed the CYLD expression in the tissues of PDX models, corresponding to each PDX strains, respectively. In accordance with our results shown in this study, immunohistochemical analysis clearly indicated that the high-sensitive strain (PDX st.1) exhibited significant lower CYLD positive rate than that of low-sensitive strain (Fig. 6E), suggesting that low CYLD expression were indeed associated with gefitinib sensitivity. Taken together, our results showed that EGFR-TKIs, such as gefitinib, could have potential to be novel therapeutic agents for the CYLD-negative OSCC patients with poor prognosis, and that CYLD expression might serve as a novel predictive biomarker for gefitinib treatment (Fig. 6F).

## Discussion

A growing body of evidence is accumulating to reveal that loss of CYLD expression in tumor tissues is closely associated with malignant transformation and poor prognosis in various malignant tumors [47]. However, despite the urgent need for novel therapeutic strategies, no clinically-effective treatments for CYLD-negative cancer patients with poor prognosis has been established.

In this study, we found that the EGFR targeted inhibitors could have potential to be novel therapeutic agents for the CYLD-negative OSCC patients with poor prognosis. As a matter of fact, most of previous studies have demonstrated that loss of CYLD expression is predominantly associated with resistance of multiple anticancer drugs [17, 23, 24, 29, 48]. In HCC, loss of CYLD expression was involved in the resistance towards treatment with doxorubicin, 5-FU, and cisplatin [17]. Other studies also reported that CYLD down-regulation caused by microRNA was associated with resistance for either gemcitabine in pancreatic cancer, and cisplatin in gastric cancer [23]. Those multiple anticancer drugs not effective for CYLD-negative cancer cells, are categorized as the cytotoxic chemotherapeutic agents, suggesting that loss of CYLD expression may also affect cell cycle progression or apoptosis [49]. Although there were a few reports suggesting the combination chemotherapy to overcome drug resistance [29, 48], it is well-known that certain severe systemic side effects are associated with combination chemotherapy [50]. Our approach identifying the crucial cell signaling pathways in CYLD-dependent malignant transformation, enabled us to develop novel therapeutic strategies utilizing EGFR targeted inhibitors as single treatment for CYLD-negative OSCC patients. EGFR-TKIs, well-established EGFR-targeted therapies in non small cell lung cancer (NSCLC) [51], are generally well-tolerated and not associated with the major side effects of chemotherapeutic agents, such as anemia, neutropenia, and thrombocytopenia [52]. Our results clearly showed that single gefitinib treatment could significantly suppressed both cell survival and EMT-like changes (Figs. 3, 4 and 5), which in turn led to markedly prolong the overall survival of CYLD-negative CDX models (Fig. 6C). In contrast to the previous report showing the gefitinib resistance caused by low-CYLD expression in NSCLC [53], it was the first repot that EGFR-TKIs were indeed effective for CYLD-negative cancer patients. In addition, our results also found that cetuximab, only EGFR-targeted inhibitor currently approved for OSCC, significantly exhibited the therapeutic effect on EMT-like changes (Fig. 5E–H). In contrast, it is noteworthy that those EGFR inhibitors-sensitive OSCC cells are indeed resistant for all chemotherapeutic agent treatment (Fig. 1). Our findings indicated that CYLD-dependent malignant transformation provided the “double-edged” aspect for treatment (resistance for chemotherapies but high sensitivity for EGFR-targeted inhibitors). More importantly, our preliminary data also found that this “double-edged” aspect was not only observed in OSCC, but also in a variety of CYLD-negative malignant cancers, such as triple negative breast cancer, ovarian cancer, and NSCLC (data not shown). Taken together, with a different perspective than combination chemotherapy to overcome drug resistance with certain side effect, our results may provide the EGFR inhibitors as novel alternative therapeutic agents for CYLD-negative cancer patients with poor prognosis.

EGFR signaling has shown to serve as a master control for cell growth and differentiation pathways in HNSCC [41]. Frequent EGFR overexpression in primary HNSCC is thought to correlate with cell survival, metastasis, and poor prognosis [54]. One of interesting findings is that CYLD knockdown may make OSCC cells transformed to EGFR signaling dependent cell survival (Fig. 3). In addition to NF-κB signaling, a well-known target of CYLD, recent studies have revealed that CYLD targeted a variety of signaling, such as, MAPK, Wnt/β-catenin, c-jun N-terminal kinase (JNK), TGF-β, and EGFR [12, 55–58]. Among these cell signaling pathways, NF-κB signaling was indeed enhanced and involved in cisplatin resistance in CYLD-knockdown OSCC cells [29], whereas NF-κB inhibitor treatment exhibited no effects on cell viability (Additional file 1: Fig. S5). Thus, EGFR signaling may predominantly play key roles in cell survival of CYLD-knockdown OSCC cells, which in turn lead to their high sensitivity to various types of EGFR inhibitors. In contrast to the previous report that deubiquitinase CYLD was necessary for proper ubiquitination and degradation of EGFR [58], our results found that CYLD knockdown significantly enhanced EGFR phosphorylation with no change of EGFR expression (Fig. 3A). Previous studies have suggested that ligand-independent EGFR endocytosis induces EGFR phosphorylation [59], and also that the involvement of CYLD in EGFR endocytosis [48], which suggests the necessity to further investigate the molecular mechanism underling high EGFR phosphorylation caused by CYLD knockdown.

Interestingly, EGFR inhibitors treatment (both gefitinib and cetuximab) also suppressed the EMT-like changes through inhibiting Smad3 phosphorylation, one of key signal molecules in TGF-β signaling (Fig. 5). Previous study reported that downregulation of CYLD promoted the invasion with EMT-like changes via TGF-β receptor I stabilization in OSCC cells [28]. Our results showed that gefitinib treatment suppressed migration and EMT-like change as effective as TGF-β specific inhibitor (Fig. 5A–D, Additional file 1: Fig. S4). Moreover, consistent with gefitinib, cetuximab treatment also suppressed both migration and EMT-like change (Fig. 5E–H). It should be noted that TGF-β signaling inhibition did not show any effect on cell viability in CYLD-knockdown OSCC cells at all (Additional file 1: Fig. S6). Because some reports revealed the cross-talk between EGFR and TGF-β signalings [60, 61], gefitinib treatment may have potential to have multiple therapeutic effects by inhibiting both signalings simultaneously with single treatment. Since the detailed molecular mechanisms underlying the CYLD dependent malignant transformation and link between EGFR and TGF-β signalings are still largely unknown, further investigation will focus on elucidating the different modes of therapeutic action in EGFR inhibitors treatment in CYLD-knockdown OSCC cells. Moreover, beyond expectations, cetuximab treatment also exhibited the inhibitory effects on the EMT-like changes (Fig. 5F–H), while cetuximab had no effect for the cell survival of CYLD-knockdown OSCC cells (Fig. 4D). The different effects on cell survival may be due to the different phosphorylation site, because gefitinib suppresses both Y1086 and Y1068 phosphorylation of EGFR, while cetuximab suppresses only Y1086 phosphorylation [62]. In addition, our preliminary experiments showing inhibitory effects of cetuximab on anchorage-independent growth, an indicator of EMT, further support the effects of cetuximab on the EMT-like changes (Additional file 1: Fig. S7). Previous study has shown that SAS cells are resistant to cetuximab in monolayer culture condition, and become sensitive in anchorage-independent culture conditions, due to unknown mechanism [63]. Since cetuximab is currently approved EGFR inhibitors for OSCC treatment, we also further investigate the molecular mechanism of cetuximab effects, in view of early clinical application.

Moreover, our finding also provides several important topics for future clinical application. The in vivo experiment using PDX models suggested that CYLD expression might serve as a novel predictive biomarker for gefitinib treatment (Fig. 6D, E). Because of significant low successful rate of drug development, especially for oncology drugs (approximately less than 5%) [64], the PDX models with patients’ clinical data, pathologies, gene profiles, are very useful tools and play important roles in preclinical studies. Thus, the PDX models are being used for drug response prediction and biomarker identification, for personalized medicine [65]. The tumor tissues of our established PDX models indeed showed the several OSCC markers (EGFR: positive, CA19: partially positive, CK(AE1/AE3): partially positive, CK13: negative), indicating that those tissues were surely originated from undifferentiated OSCC tissue (Additional file 1: Fig. S8). Our results showed that low CYLD expression were indeed associated with gefitinib sensitivity in this PDX models, suggesting that molecular diagnostics targeting CYLD expression may be useful for prediction and validation of gefitinib response (Fig. 6F). Although the past Phase III study of gefitinib did not show significant effects in HNSCC [66], it could be possible that prospective clinical trial applying patient stratification by CYLD expression may improve the outcome from gefitinib therapy. It should be noted that, although low CYLD expression were indeed associated with gefitinib sensitivity, there were still 5–25% of CYLD-positive cells (Fig. 6E). While, it is noteworthy that the chemotherapeutic agents currently approved for standard treatment for OSCC patients was indeed effective for CYLD-positive cells (Fig. 1). Therefore, in view of treating the patients with mix population of CYLD-positive and negative, in addition to gefitinib treatment, neoadjuvant or adjuvant therapy by chemotherapeutic agents, such as cisplatin or 5-FU, may be effective for CYLD-positive cells. Another interesting finding is that, in addition to gefitinib, various types of EGFR-TKIs, such as erlotinib, afatinib, and osimertinib, were also effective for CYLD-knockdown OSCC cells (Fig. 4A–C). It has been shown that the drug response of first (gefitinib, erlotinib), second (afatinib), and third (osimertinib) generation of EGFR-TKI is varied depending on structural change in ATP binding site of EGFR by mutation [67]. Because no mutation responsible for EGFR-TKIs effects was detected in OSCC cells used in this study [68], the structural changes of EGFR caused by CYLD knockdown might allow all EGFR-TKIs to bind and show therapeutic effects. While the detailed mechanism is still unknown, further investigation may have chance to provide more therapeutic option for the treatment of CYLD-negative OSCC patients in the future.

## Conclusions

In summary, this study provide evidence for potential use of EGFR-targeted molecular therapies for CYLD-knockdown OSCC cells with chemoresistance. Our experimental evidence showed that EGFR-TKIs, such as gefitinib, was effective for inhibiting both cell survival and EMT-like changes in CYLD-knockdown OSCC cells. In addition, we also found that cetuximab, the approved drug for OSCC, significantly suppressed the EMT-like changes. Moreover, molecular diagnostics targeting CYLD expression might serve as a novel predictive biomarker for gefitinib treatment. Further clinical studies focusing on the efficacy and safety of these EGFR-targeted molecular therapies will provide novel opportunities for the treatment of for CYLD-negative OSCC patients with poor prognosis.

## Supplementary Information

**Additional file 1: Figure S1.** CYLD-knockdown by CYLD-specific siRNA at mRNA level. **Figure S2.** The proteins involved in Akt and ERK signaling pathway in CYLD-negative OSCC cells**. Figure S3.** Cell survival rate in CYLD-knockdown OSCC cells by different siCYLD #2 and in other OSCC cells. **Figure S4.** Gefitinib treatment increased the expression of E-cadherin, an EMT biomarker. **Figure S5.** NF-κB signaling pathway does not participate in cell survival in CYLD-negative OSCC cells. **Figure S6.** TGF-β signaling pathway does not participate in cell survival in CYLD-negative OSCC cells. **Figure S7.** Cetuximab treatment suppressed anchorage-independent growth of CYLD-negative OSCC cells. **Figure S8.** Immunohistochemical analysis for OSCC marker expression in tumor tissues from PDX models (PDX st.1 & PDX st.2).

## Data Availability

The datasets used and/or analyzed during the current study are available from the corresponding author on reasonable request.

## Abbreviations

CYLD: Cylindromatosis
NF-κB: Nuclear Factor-kappa B
OSCC: Oral squamous cell carcinoma
EMT: Epithelial-mesenchymal transition
EGFR: Epidermal Growth factor receptor
EGFR-TKI: EGFR tyrosine kinase inhibitor
siRNA: Small interfering RNA
RT-qPCR: Quantitative real-time PCR
ERK: Extracellular signal-regulated kinase.
CDX: Cell line-derived xenograft
PDX: Patient-derived xenograft

## References

[1] Bignell GR, Warren W, Seal S, Yang S, Wang HY, Chen JJ (2000). Identification of the familial cylindromatosis tumour-suppressor gene. Nat Genet.

[2] Sun S-C (2010). CYLD: a tumor suppressor deubiquitinase regulating NF-κB activation and diverse biological processes. Cell Death Differ.

[3] Trompouki E, Hatzivassiliou E, Tsichritzis T, Farmer H, Ashworth A, Mosialos G (2003). CYLD is a deubiquitinating enzyme that negatively regulates NF-κB activation by TNFR family members. Nature.

[4] Brummelkamp TR, Nijman SMB, Dirac AMG, Bernards R (2003). Loss of the cylindromatosis tumour suppressor inhibits apoptosis by activating NF-κB. Nature.

[5] Kovalenko A, Chable-Bessia C, Cantarella G, Israël A, Wallach D, Courtois G (2003). The tumour suppressor CYLD negatively regulates NF-κB signalling by deubiquitination. Nature.

[6] Jono H, Lim JH, Chen L-F, Xu H, Trompouki E, Pan ZK (2004). NF-κB is essential for induction of CYLD, the negative regulator of NF-κB. J Biol Chem.

[7] Yoshida H, Jono H, Kai H, Li J-D (2005). The tumor suppressor Cylindromatosis (CYLD) acts as a negative regulator for toll-like receptor 2 signaling via negative cross-talk with TRAF6 and TRAF7. J Biol Chem.

[8] Sakai A, Koga T, Lim J-H, Jono H, Harada K, Szymanski E (2007). The bacterium, nontypeable *Haemophilus influenzae*, enhances host antiviral response by inducing Toll-like receptor 7 expression. FEBS J.

[9] Lim JH, Stirling B, Derry J, Koga T, Jono H, Woo CH (2007). Tumor suppressor CYLD regulates acute lung injury in lethal *Streptococcus pneumoniae* infections. Immunity.

[10] Lim JH, Jono H, Koga T, Woo CH, Ishinaga H, Bourne P (2007). Tumor suppressor CYLD acts as a negative regulator for non-typeable *Haemophilus influenza*-induced inflammation in the middle ear and lung of mice. PLoS ONE.

[11] Koga T, Lim JH, Jono H, Ha UH, Xu H, Ishinaga H (2008). Tumor suppressor cylindromatosis acts as a negative regulator for *Streptococcus pneumoniae*-induced NFAT signaling. J Biol Chem.

[12] Lim JH, Jono H, Komatsu K, Woo CH, Lee J, Miyata M (2012). CYLD negatively regulates transforming growth factor-β-signalling via deubiquitinating Akt. Nat Commun.

[13] Komatsu K, Lee J-Y, Miyata M, Hyang Lim J, Jono H, Koga T (2013). Inhibition of PDE4B suppresses inflammation by increasing expression of the deubiquitinase CYLD. Nat Commun.

[14] Courtois G, Gilmore TD (2006). Mutations in the NF-κB signaling pathway: implications for human disease. Oncogene.

[15] Massoumi R, Kuphal S, Hellerbrand C, Haas B, Wild P, Spruss T (2009). Down-regulation of CYLD expression by Snail promotes tumor progression in malignant melanoma. J Exp Med.

[16] Kinoshita H, Okabe H, Beppu T, Chikamoto A, Hayashi H, Imai K (2013). CYLD downregulation is correlated with tumor development in patients with hepatocellular carcinoma. Mol Clin Oncol.

[17] Urbanik T, Köhler BC, Boger RJ, Wörns MA, Heeger S, Otto G (2011). Down-regulation of CYLD as a trigger for NF-κB activation and a mechanism of apoptotic resistance in hepatocellular carcinoma cells. Int J Oncol.

[18] Ishikawa Y, Tsunoda K, Shibazaki M, Takahashi K, Akasaka T, Masuda T (2012). Downregulation of cylindromatosis gene, CYLD, confers a growth advantage on malignant melanoma cells while negatively regulating their migration activity. Int J Oncol.

[19] Kuphal S, Shaw-Hallgren G, Eberl M, Karrer S, Aberger F, Bosserhoff AK (2011). GLI1-dependent transcriptional repression of CYLD in basal cell carcinoma. Oncogene.

[20] Hayashi M, Jono H, Shinriki S, Nakamura T, Guo J, Sueta A (2014). Clinical significance of CYLD downregulation in breast cancer. Breast Cancer Res Treat.

[21] Guo J, Shinriki S, Su Y, Nakamura T, Hayashi M, Tsuda Y (2014). Hypoxia suppresses cylindromatosis (CYLD) expression to promote inflammation in glioblastoma: possible link to acquired resistance to anti-VEGF therapy. Oncotarget.

[22] Miyake S, Miwa T, Yoneda G, Kanemaru A, Saito H, Minoda R (2020). Relationship between clinicopathological characteristics and CYLD expression in patients with cholesteatoma. PLoS ONE.

[23] Takiuchi D, Eguchi H, Nagano H, Iwagami Y, Tomimaru Y, Wada H (2013). Involvement of microRNA-181b in the gemcitabine resistance of pancreatic cancer cells. Pancreatology.

[24] Zhu M, Zhou X, Du Y, Huang Z, Zhu J, Xu J (2016). miR-20a induces cisplatin resistance of a human gastric cancer cell line via targeting CYLD. Mol Med Rep.

[25] Warnakulasuriya S (2009). Global epidemiology of oral and oropharyngeal cancer. Oral Oncol.

[26] Jimenez L, Jayakar SK, Ow TJ, Segall JE (2015). Mechanisms of invasion in head and neck cancer. Arch Pathol Lab Med.

[27] Pignon JP, Bourhis J, Domenge C, Designé L (2000). Chemotherapy added to locoregional treatment for head and neck squamous-cell carcinoma: three meta-analyses of updated individual data. Lancet.

[28] Shinriki S, Jono H, Maeshiro M, Nakamura T, Guo J, Li JD (2018). Loss of CYLD promotes cell invasion via ALK5 stabilization in oral squamous cell carcinoma. J Pathol.

[29] Suenaga N, Kuramitsu M, Komure K, Kanemaru A, Takano K, Ozeki K (2019). Loss of tumor suppressor CYLD expression triggers cisplatin resistance in oral squamous cell carcinoma. Int J Mol Sci.

[30] Masuda T, Saito N, Tomita M, Ishihama Y (2009). Unbiased quantitation of *Escherichia coli* membrane proteome using phase transfer surfactants. Mol Cell Proteomics.

[31] Masuda T, Tomita M, Ishihama Y (2008). Phase transfer surfactant-aided trypsin digestion for membrane proteome analysis. J Proteome Res.

[32] Rappsilber J, Mann M, Ishihama Y (2007). Protocol for micro-purification, enrichment, pre-fractionation and storage of peptides for proteomics using StageTips. Nat Protoc.

[33] Rappsilber J, Ishihama Y, Mann M (2003). Stop and go extraction tips for matrix-assisted laser desorption/ionization, nanoelectrospray, and LC/MS sample pretreatment in proteomics. Anal Chem.

[34] Gillet LC, Navarro P, Tate S, Röst H, Selevsek N, Reiter L (2012). Targeted data extraction of the MS/MS spectra generated by data-independent acquisition: a new concept for consistent and accurate proteome analysis. Mol Cell Proteomics.

[35] Okada S, Vaeteewoottacharn K, Kariya R (2019). Application of highly immunocompromised mice for the establishment of patient-derived xenograft (PDX) models. Cells.

[36] Vaeteewoottacharn K, Pairojkul C, Kariya R, Muisuk K, Imtawil K, Chamgramol Y (2019). Establishment of highly transplantable cholangiocarcinoma cell lines from a patient-derived xenograft mouse model. Cells.

[37] Zhong LP, Zhang CP, Ren GX, Guo W, William WN, Sun J (2013). Randomized phase III trial of induction chemotherapy with docetaxel, cisplatin, and fluorouracil followed by surgery versus up-front surgery in locally advanced resectable oral squamous cell carcinoma. J Clin Oncol.

[38] Ai D, Chen Y, Liu Q, Zhang J, Deng J, Zhu H (2018). Comparison of paclitaxel in combination with cisplatin (TP), carboplatin (TC) or fluorouracil (TF) concurrent with radiotherapy for patients with local advanced oesophageal squamous cell carcinoma: a three-arm phase III randomized trial (ESO-Shanghai 2). BMJ Open.

[39] Peng Y, Wang Y, Zhou C, Mei W, Zeng C (2022). PI3K/Akt/mTOR pathway and its role in cancer therapeutics: are we making headway?. Front Oncol.

[40] Barbosa R, Acevedo LA, Marmorstein R (2021). The MEK/ERK network as a therapeutic target in human cancer. Mol Cancer Res.

[41] Cai J, Sun M, Ge X, Sun Y (2017). EGFR tyrosine kinase inhibitors differentially affect autophagy in head and neck squamous cell carcinoma. Biochem Biophys Res Commun.

[42] Mitsudomi T, Morita S, Yatabe Y, Negoro S, Okamoto I, Tsurutani J (2010). Gefitinib versus cisplatin plus docetaxel in patients with non-small-cell lung cancer harbouring mutations of the epidermal growth factor receptor (WJTOG3405): an open label, randomised phase 3 trial. Lancet Oncol.

[43] Yang Z, Hackshaw A, Feng Q, Fu X, Zhang Y, Mao C (2017). Comparison of gefitinib, erlotinib and afatinib in non-small cell lung cancer: a meta-analysis. Int J Cancer.

[44] Yamaoka T, Ohba M, Ohmori T (2017). Molecular-targeted therapies for epidermal growth factor receptor and its resistance mechanisms. Int J Mol Sci.

[45] Bonner JA, Harari PM, Giralt J, Cohen RB, Jones CU, Sur RK (2010). Radiotherapy plus cetuximab for locoregionally advanced head and neck cancer: 5-year survival data from a phase 3 randomised trial, and relation between cetuximab-induced rash and survival. Lancet Oncol.

[46] Pompili L, Porru M, Caruso C, Biroccio A, Leonetti C (2016). Patient-derived xenografts: a relevant preclinical model for drug development. J Exp Clin Cancer Res.

[47] Miyake S, Kanemaru A, Saito H, Jono H (2021). CYLD: a novel stratification marker for malignant tumors. J Asian Assoc Sch Pharm.

[48] Liu R, Shinriki S, Maeshiro M, Hirayama M, Jono H, Yoshida R (2021). The tumour suppressor cyld is required for clathrin-mediated endocytosis of EGFR and cetuximab-induced apoptosis in head and neck squamous cell carcinoma. Cancers (Basel).

[49] Cao X, Hou J, An Q, Assaraf YG, Wang X (2020). Towards the overcoming of anticancer drug resistance mediated by p53 mutations. Drug Resist Updat.

[50] Carrick S, Parker S, Thornton CE, Ghersi D, Simes J, Wilcken N (2009). Single agent versus combination chemotherapy for metastatic breast cancer. Cochrane Database Syst Rev.

[51] Giaccone G (2005). Epidermal growth factor receptor inhibitors in the treatment of non-small-cell lung cancer. J Clin Oncol.

[52] Liu S, Wang D, Chen B, Wang Y, Zhao W, Wu J (2011). The safety and efficacy of EGFR TKIs monotherapy versus single-agent chemotherapy using third-generation cytotoxics as the first-line treatment for patients with advanced non-small cell lung cancer and poor performance status. Lung Cancer.

[53] Yuan Y, Liu L, Wang Y, Liu S (2020). Reduced expression of CYLD promotes cell survival and inflammation in gefitinib-treated NSCLC PC-9 cells: targeting CYLD may be beneficial for acquired resistance to gefitinib therapy. Cell Biol Int.

[54] Network CGA (2015). Comprehensive genomic characterization of head and neck squamous cell carcinomas. Nature.

[55] Tesio M, Tang Y, Müdder K, Saini M, von Paleske L, Macintyre E (2015). Hematopoietic stem cell quiescence and function are controlled by the CYLD–TRAF2–p38MAPK pathway. J Exp Med.

[56] Tauriello DV, Haegebarth A, Kuper I, Edelmann MJ, Henraat M, Canninga-van Dijk MR (2010). Loss of the tumor suppressor CYLD enhances Wnt/β-catenin signaling through K63-linked ubiquitination of Dvl. Mol Cell.

[57] Reiley W, Zhang M, Sun S-C (2004). Negative regulation of JNK signaling by the tumor suppressor CYLD. J Biol Chem.

[58] Sanchez-Quiles V, Akimov V, Osinalde N, Francavilla C, Puglia M, Barrio-Hernandez I (2017). Cylindromatosis tumor suppressor protein (CYLD) deubiquitinase is necessary for proper ubiquitination and degradation of the epidermal growth factor receptor. Mol Cell Proteomics.

[59] Tomas A, Vaughan SO, Burgoyne T, Sorkin A, Hartley JA, Hochhauser D (2015). WASH and Tsg101/ALIX-dependent diversion of stress-internalized EGFR from the canonical endocytic pathway. Nat Commun.

[60] Kamaraju AK, Roberts AB (2005). Role of Rho/ROCK and p38 MAP kinase pathways in transforming growth factor-β-mediated smad-dependent growth Inhibition of human breast carcinoma cells in Vivo. J Biol Chem.

[61] Liu Q, Zhang Y, Mao H, Chen W, Luo N, Zhou Q (2012). A crosstalk between the Smad and JNK signaling in the TGF-β-induced epithelial-mesenchymal transition in rat peritoneal mesothelial cells. PLoS ONE.

[62] Matsumoto Y, Sakurai H, Kogashiwa Y, Kimura T, Matsumoto Y, Shionome T (2017). Inhibition of epithelial–mesenchymal transition by cetuximab via the EGFR-GEP100-Arf6-AMAP1 pathway in head and neck cancer. Head Neck.

[63] Ohnishi Y, Yasui H, Kakudo K, Nozaki M (2015). Cetuximab-resistant oral squamous cell carcinoma cells become sensitive in anchorage-independent culture conditions through the activation of the EGFR/AKT pathway. Int J Oncol.

[64] DiMasi JA, Reichert JM, Feldman L, Malins A (2013). Clinical approval success rates for investigational cancer drugs. Clin Pharmacol Ther.

[65] Hidalgo M, Amant F, Biankin AV, Budinská E, Byrne AT, Caldas C (2014). Patient-derived Xenograft models: an emerging platform for translational cancer research. Cancer Discov.

[66] Stewart JS, Cohen EE, Licitra L, Van Herpen CM, Khorprasert C, Soulieres D (2009). Phase III study of gefitinib compared with intravenous methotrexate for recurrent squamous cell carcinoma of the head and neck. J Clin Oncol.

[67] Robichaux JP, Le X, Vijayan RSK, Hicks JK, Heeke S, Elamin YY (2021). Structure-based classification predicts drug response in EGFR-mutant NSCLC. Nature.

[68] Nakamura Y, Togashi Y, Nakahara H, Tomida S, Banno E, Terashima M (2016). Afatinib against esophageal or head-and-neck squamous cell carcinoma: significance of activating oncogenic HER4 mutations in HNSCC. Mol Cancer Ther.

