# Supplementary material for: Potential use of EGFR-targeted molecular therapies for tumor suppressor CYLD-negative and poor prognosis oral squamous cell carcinoma with chemoresistance
Source: Cancer Cell Int. 2022 Nov 15;22:358. doi: 10.1186/s12935-022-02781-x (PMC9664721; doi:10.1186/s12935-022-02781-x)
Supplement: Supplementary file 1 — Additional file 1: Figure S1. CYLD-knockdown by CYLD-specific siRNA at mRNA level. Figure S2. The proteins involved in Akt and ERK signaling pathway in CYLD-negative OSCC cells. Figure S3. Cell survival rate in CYLD-knockdown OSCC cells by different siCYLD #2 and in other OSCC cells. Figure S4. Gefitinib treatment increased the expression of E-cadherin, an EMT biomarker. Figure S5. NF-κB signaling pathway does not participate in cell survival in CYLD-negative OSCC cells. Figure S6. TGF-β signaling pathway does not participate in cell survival in CYLD-negative OSCC cells. Figure S7. Cetuximab treatment suppressed anchorage-independent growth of CYLD-negative OSCC cells. Figure S8. Immunohistochemical analysis for OSCC marker expression in tumor tissues from PDX models (PDX st.1 & PDX st.2). [file 12935_2022_2781_MOESM1_ESM.docx]

**Supplementary Figure 1**


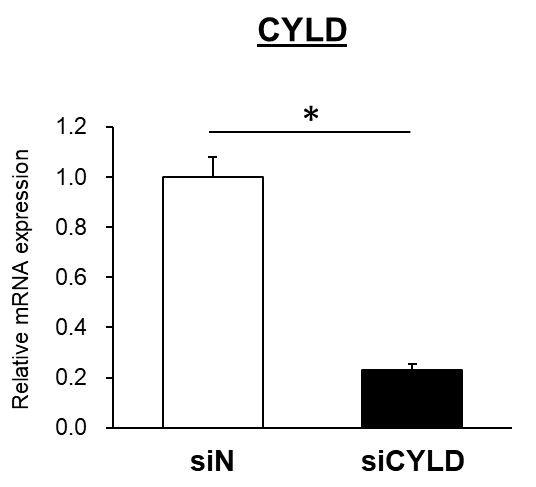


**Supplementary Figure 1. CYLD-knockdown by CYLD-specific siRNA at mRNA level.**

SAS cells were transfected with control siRNA (siN) or CYLD-specific siRNA (siCYLD) for 48 h, and CYLD mRNA expression was assessed by RT-qPCR. Values are means ± S.D. of triplicate samples. * p < 0.05, in Student's t-test.

**Supplementary Figure 2**

**Supplementary Figure 2.** **The proteins involved in Akt and ERK signaling pathway in CYLD-negative OSCC cells.** In proteome analysis, the proteins involved in PI3K-Akt pathway (LAMB3, ITGB4, ITGA2, GNB1, LAMC2, ITGA6, and YWHAH) were pointed as yellow square, and the proteins involved in ERK pathway (RALB, IL1B) were pointed as blue square.

**Supplementary Figure 3**

**Supplementary Figure 3.** **Cell survival rate in CYLD-knockdown OSCC cells by different siCYLD #2 and in other OSCC cells.** SAS cells were transfected with siN and siCYLD #2 (sense: 5’-rGrArArCrGrAUrGUrArGrArAUrAUUrAUTT-3’; antisense: 5’-rAUrArAUrAUUrCUrArCrAUrCrGUUrCTT-3’) and treated with cisplatin (2.5µg/mL, A) and gefitinib (10 µM, B). Gefitinib was also significantly effective in other CYLD-knockdown OSCC cells, such as, HSC3 (C) and NA cells (D). Cell survival rate was assessed after 72 h from treatment. Values are means ± S.D. of triplicate samples. * p < 0.05, ** p < 0.01 in Tukey-Kramer method.

**Supplementary Figure 4**

**Supplementary Figure 4.** **Gefitinib treatment increased the expression of E-cadherin, an EMT biomarker.** SAS cells were transfected with siN or siCYLD and treated with 10 μM gefitinib. The expression of E-cadherin, an epithelial marker, was assessed by immunoblotting.

**Supplementary Figure 5**

**Supplementary Figure 5.** **NF-κB signaling pathway does not participate in cell survival in CYLD-negative OSCC cells.** OSCC cells were treated with BAY11-7085 (NF-κB signaling inhibitor, 10 µM). Cell survival rate was assessed after 72 h. Values are means ± S.D. of triplicate samples. n.s.: not significant.

**Supplementary Figure 6**

**Supplementary Figure 6.** **TGF-β signaling pathway does not participate in cell survival in CYLD-negative OSCC cells.** OSCC cells were treated with LY2157299 (TGF-β signaling inhibitor, 1-100 µM). Cell survival rate was assessed after 48 h. Values are means ± S.D. of triplicate samples. n.s; not significant in Tukey-Kramer method.

**Supplementary Figure 7**

**Supplementary Figure 7.** **Cetuximab treatment suppressed anchorage-independent growth of CYLD-negative OSCC cells.** OSCC cells were plated onto 6-well plates and incubated at 37℃, When the cells grew to colonies, the colonies were washed with ice cold PBS and fixed with 100% methanol. Cells were stained by crystal violet, and the ratio of the stained area was quantified by Image J software. Values are means ± SD of triplicate samples. * p < 0.05, ** p < 0.01 in Tukey-Kramer method.

**Supplementary Figure 8**

**Supplementary Figure 8.** **Immunohistochemical analysis for OSCC marker expression in tumor tissues from PDX models (PDX st.1 & PDX st.2).** Tumor tissues from PDX models showed several OSCC markers (EGFR: positive, CA19: partially positive, CK(AE1/AE3): partially positive, CK13: negative), indicating that those tissues were surely originated from undifferentiated OSCC tissue. Scale bars show 50 µm.
